# The landscape of cell surface target expression in treatment resistant early-stage breast cancer

**DOI:** 10.1007/s10549-026-08120-2

**Published:** 2026-11-09

**Authors:** Per Karlsson, Marina N. Sharifi, Erik Holmberg, Hamza Bakhtiar, Emma Niméus, Fredrika Killander, Martin Sjöström, Shuang G. Zhao

**Affiliations:** 1 Department of Oncology, Institute of Clinical Sciences, University of Gothenburg, Gothenburg, Sweden; 2 Department of Medicine, University of Wisconsin, Madison, WI USA; 3 Department of Radiation Medicine, University of Wisconsin, Madison, WI USA; 4 Institute of Clinical Sciences, Lund University, Lund, Sweden; 5 Department of Surgery, Skåne University Hospital, Malmö, Sweden; 6 Department of Haematology, Oncology, Radiations Physics, Skåne University Hospital, Lund, Sweden; 7 Department of Urology, University of California, San Francisco, CA USA; 8 University of Texas MD Anderson Cancer Center, Houston, TX USA

**Keywords:** Breast cancer, Cell surface targets

## Abstract

**Purpose:**

Disease recurrence after definitive therapy remains a major cause of morbidity and mortality in early-stage breast cancer, with limited options for treatment intensification. Cell surface–targeted (CST) therapies, including antibody-drug conjugates (ADCs), radiopharmaceuticals, and immune-based approaches, have demonstrated success in treating malignancies. However, the known CST repertoire in breast cancer is narrow, largely restricted to HER2 and TROP2, highlighting an unmet need for novel targets in treatment-resistant disease.

**Methods:**

We profiled CST expression using large-scale gene expression datasets annotated with clinical outcomes, including SCAN-B (*n* ≈ 10,000), aggregated neoadjuvant chemotherapy cohorts (*n* ≈ 4,000), and the randomized SWEBCG91RT trial (*n* = 765). CSTs with therapies in clinical trials were prioritized. RNA-protein correlation was assessed using CPTAC and SCMP datasets. Associations with recurrence-free survival (RFS) and treatment response were analyzed using Cox regression and Fisher’s exact test with Benjamini-Hochberg correction.

**Results:**

Of 78 CSTs under active clinical investigation, RNA expression strongly predicted protein expression (median accuracy 80–94%). In ER+ breast cancer treated with endocrine therapy, nine CSTs (including *CEACAM5/6*, *EPCAM*, *NECTIN4*) were associated with significantly worse RFS. Of these, *EPCAM* and *CEACAM5* were also associated with poor outcomes specifically after radiotherapy in the SWEBCG91RT trial. In HER2 + disease, high expression of *FOLH1* and *TNFRSF10B* predicted relapse despite anti-HER2 therapy. In triple-negative breast cancer, six CSTs were associated with residual disease after neoadjuvant therapy.

**Conclusions:**

These analyses identify potential candidates for repositioning CST drugs, and single-cell analysis revealed substantial intratumoral heterogeneity for several targets, informing payload selection strategies. CSTs associated with treatment resistance across breast cancer subtypes represent actionable opportunities for therapeutic repositioning. These findings provide a framework for rational integration of CST therapies in early-stage breast cancer.

**Supplementary Information:**

The online version contains supplementary material available at https://doi.org/10.1007/s10549-026-08120-2.

## Introduction

Disease recurrence remains a significant clinical challenge in the treatment of early-stage breast cancer, occurring in up to a third of patients who are disease free after completion of definitive therapy [9, 39] and driving breast cancer morbidity and mortality. Despite advances in neoadjuvant and adjuvant therapy strategies across breast cancer subtypes, patients with adverse prognostic features remain at high risk of metastatic and life limiting recurrence, which carries a 5-year survival rate of just 30% [17]. Consequently, there is an unmet need to identify new therapeutic approaches for treatment intensification for this group of patients.

Cell surface–targeted (CST) therapies have emerged as a transformative class of anti-cancer agents across both hematologic and solid tumors. Unlike traditional cytotoxic chemotherapies, these therapies leverage the differential expression of surface proteins on cancer cells to enhance tumor specificity and reduce systemic toxicity. Several therapeutic modalities have been developed to exploit these targets, each with distinct mechanisms of action. Among them, Antibody-Drug Conjugates (ADCs) have shown remarkable clinical success by delivering potent cytotoxic agents directly to tumor cells via monoclonal antibodies. In addition to ADCs, other FDA-approved strategies include Radiopharmaceutical Therapies (RPTs), which deliver therapeutic radionuclides to tumor sites, and a growing array of immune-based approaches such as Chimeric Antigen Receptor (CAR) T cells (and CAR-NK, CAR-dendritic cells, CAR-macrophages) and Bi-specific T-cell Engagers (BiTEs).

ADCs found early success in breast cancer, including the first FDA approval for this drug class. There are now four FDA approved ADCs in breast cancer spanning both localized and metastatic disease, all of which target either HER2 or TROP2, two cell surface proteins with high expression in breast cancer, but also relatively broad expression in normal tissues (see Figure S7 from https://insight.jci.org/articles/view/183674/sd/3) [43]. While currently approved ADCs in breast cancer are beneficial, challenges still remain. The limited cell surface target repertoire and lack of tumor cell specificity for currently FDA approved ADCs in breast cancer leads to challenges related to both toxicity profile and treatment sequencing. Numerous CST agents targeting diverse surface proteins are currently under clinical investigation across solid tumors, leading to potential opportunities to re-position CST therapies from other cancers to address the need for novel approaches in treatment-resistant breast cancer. To identify high value candidate agents already in human development for repositioning, we took a clinically informed approach to identify CSTs with enriched target expression in treatment-resistant early-stage breast cancers, utilizing breast cancer gene expression datasets annotated with clinical outcomes derived from several large clinical cohorts [5, 46] and a randomized phase III trial [44, 45].

## Methods

### Datasets

A list of cell surface targets with therapies in clinical trials and their expression levels across normal tissues was published previously [43]. To investigate RNA-protein expression correlation, we utilized paired RNA and protein expression data from primary tumors from the CPTAC (pdc.cancer.gov/pdc/cptac-pancancer), and cell lines from the SCMP [18]. All samples were used, as this was intended to broadly interrogate the association between high RNA and protein expression [43]. Three published clinical breast cancer cohorts with gene expression profiling were interrogated. The SCAN‑B (Sweden Cancerome Analysis Network – Breast) initiative is a large-scale, population-based study with nearly 10,000 available patients with long-term clinical outcomes [46] (Table S1). We also utilized an aggregated cohort of multiple neoadjuvant studies totaling nearly 4,000 available patients [5]. Available demographic data can be found in the original publications. Finally, we utilized the randomized Phase III SWEBCG91RT trial of adjuvant radiotherapy in early-stage breast cancer with 765 available patients [44, 45] (demographic data can be found in the original publications). Subsets based on receptor status were then identified in these datasets. To investigate the heterogeneity of CST expression in breast cancer, we utilized the Curated Cancer Cell Atlas of Tumors [14] as previously described [43]. Ethics declaration: not applicable.

### Analytical methods

Processing of each dataset was performed as described in the original citations. For most genes, there is no universally accepted biologically relevant expression threshold that clearly distinguishes high from low expression in breast cancer. We selected the upper quartile as the “high-expression” group. Defining high expression as the top 25% identifies a sufficiently large patient population to support the potential development and evaluation of CST-targeted therapeutic strategies in future clinical studies. In each of the clinical breast cancer datasets, we sought to focus on only CSTs with high expression, and CSTs with low expression in the majority of samples were not analyzed. For each CST, this was defined that the 25th percentile of CST expression had to be greater than the median of all genes in the cohort. Gene expression was scaled in each sample from 0 to 1 in each sample so that values were human-interpretable and comparable across samples/assays. For example, a value of 0.85 for a gene X in a sample would indicate that 85% of all genes were expressed lower than gene X, and 15% were expressed higher. Cox regression was used for time-to-event data, and Fisher’s exact test was used for categorical data. Multiple testing correction was performed using the p.adjust R function and the Benjamini Hochberg (BH) method. A p-value of 0.05 was used to determine significance. All tests were two-sided.

## Results

### CSTs under clinical investigation

**Fig. 1:**
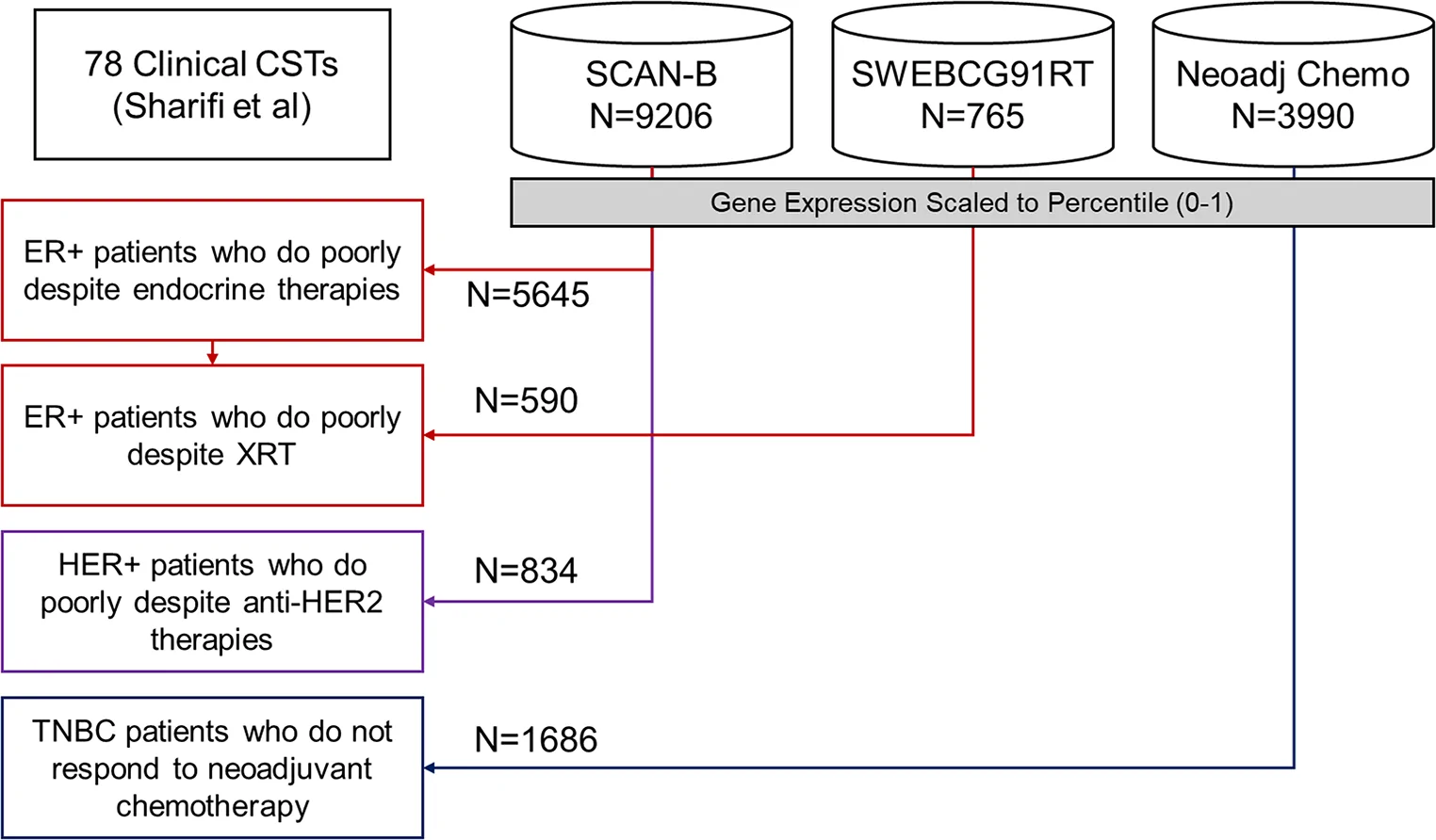
Schematic of datasets and patients

We utilized a published list of interventional CST clinical trials and their targets in adult solid tumors [43], which identified a total of 78 CSTs with therapies in active development (Fig. 1). In general, the CSTs are not highly inter-correlated (Figure S2). Using clinical cancer samples from the Clinical Proteomic Tumor Analysis Consortium (CPTAC), we found that these targets demonstrated strong concordance between high RNA expression (defined as being in the top quartile) compared to high protein expression (similarly defined). The median accuracy of high RNA predicting high protein expression across these CSTs was 80% (Figure S1A). Similarly, in cell lines from the Sanger Cell Model Passports (SCMP) [18], the median accuracy of high RNA predicting high protein expression across cell surface targets was 94% (Figure S1B), suggesting that high RNA expression can be used to accurately infer high protein expression.

### CSTs expressed in ER+ tumors at high risk of relapse after endocrine therapy

**Fig. 2:**
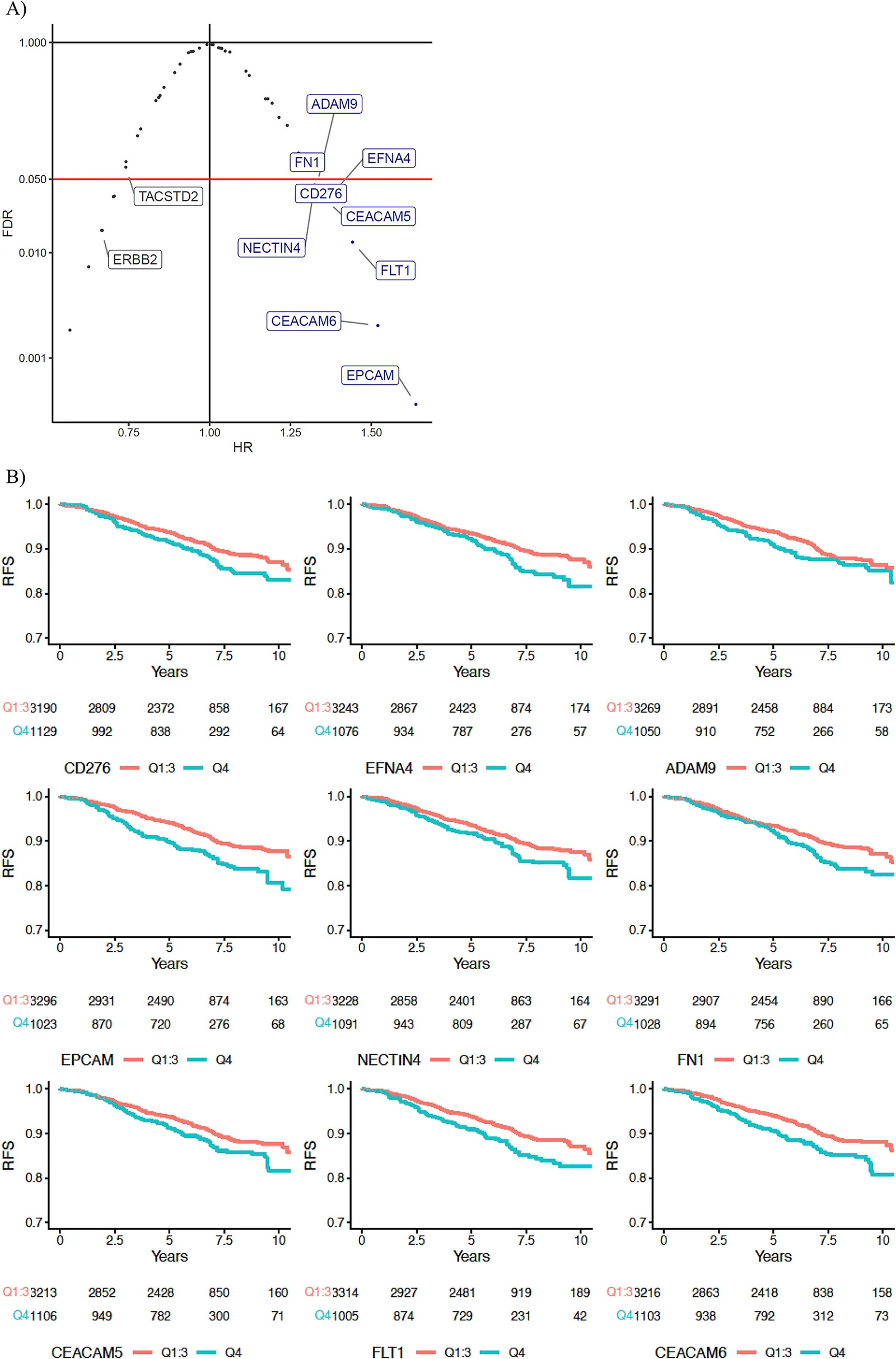
SCAN-B, ER+ treated with endocrine therapy (*N* = 5645). Volcano plot of the FDR vs. HR (**A**) and Kaplan-Meier curves (**B**) of top quartile (Q4) vs. bottom three quartiles (Q1-3) for each CST. FDR: False discovery rate, HR: hazard ratio, RFS: recurrence-free survival

In early-stage ER+ breast cancer, adjuvant endocrine therapy significantly improves survival, but life limiting metastatic recurrences still occur in up to 10% of lower risk cancers, and as high as 40% of higher risk cancers over the 20 years after initial diagnosis [30]. We examined expression of the 78 CSTs under active development across 5,645 patients with ER+/HER2- breast cancer who received endocrine therapy in the SCAN-B cohort (Table S2). To identify CSTs expressed in ER+ tumors at higher risk of relapse after endocrine therapy, we evaluated the association between CST expression and recurrence-free survival (RFS) in this cohort. High CST expression was defined as the top quartile per above. Interestingly, expression of both *ERBB2* (HER2) and *TACSTD2* (TROP2), the targets for existing FDA-approved ADCs in breast cancer, were associated with better RFS (Fig. 2A). While HER2 + breast cancer is historically associated with more aggressive disease, the clinical trajectory has improved dramatically with the use of targeted therapies [27, 52]. After multiple testing correction, we identified nine CSTs in which high expression was significantly associated with worse recurrence-free survival (RFS) (Fig. 2B). The majority of these remained significantly associated after adjusting for clinicopathologic variables (age, nodal status, grade, pT-stage, PAM50; Table S3). Protein expression of most of these CSTs has been identified in ER+ luminal breast cancers (including *CD276* [24], *EPCAM* [1, 29, 32], *CEACAM5* [7, 36], *NECTIN4* [21, 35], *FLT1* [23], *FN1* [4, 48, 49], and *CEACAM6* [6, 37, 51]). Protein expression of several of these CSTs has previously been shown to be associated with poor prognosis in ER+ breast cancers including EPCAM [42], NECTIN4 [21, 35], CEACAM5 [7, 36], and CEACAM6 [12] supporting the validity of our approach to identify CSTs expressed in tumors with higher risk of relapse. High CEACAM6 expression has been specifically implicated in endocrine resistance in pre-clinical models of ER+ breast cancer and [25, 26] higher risk of relapse after tamoxifen treatment in patient tumors [26]. Expression of FN1 in breast cancer extracellular matrix is also associated with tamoxifen resistance [34, 38, 49, 56]. Taken together, this demonstrates that we have identified a set of CSTs associated with higher risk of relapse after endocrine therapy encompassing both known and novel targets, all of which have therapies in development or FDA approved that could be repositioned to this patient population to potentially improve outcomes, particularly in the patients with the highest risk of relapse.

### CSTs expressed in ER+ tumors at high risk of relapse after radiotherapy

**Fig. 3:**
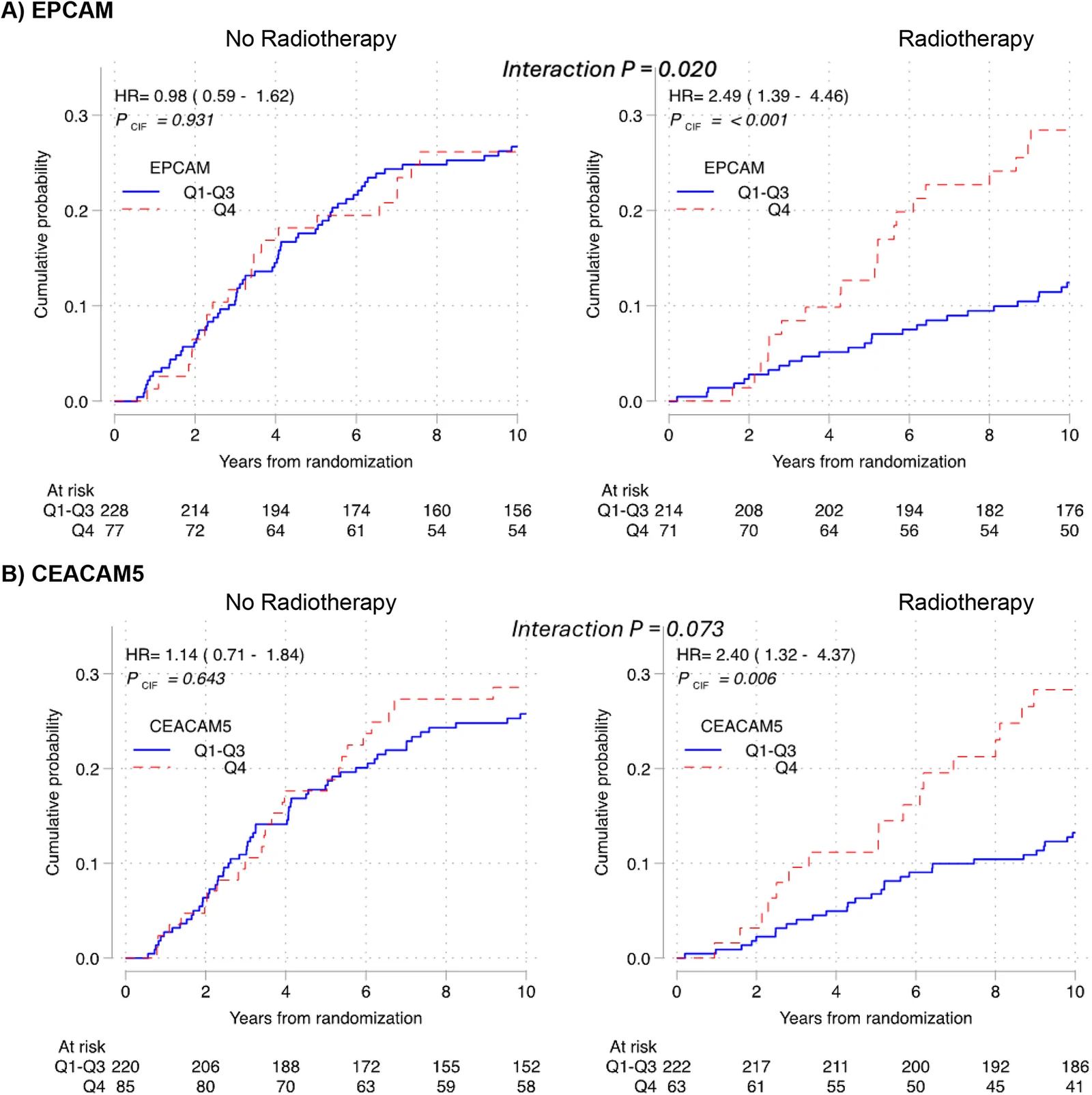
SWEBCG91RT, ER + IHC (*N* = 590). Kaplan-Meier curves of top quartile (Q4) vs. bottom three quartiles (Q1-3) in the No Radiotherapy and Radiotherapy arms for EPCAM (**A**) and CEACAM5 (**B**)

Adjuvant radiotherapy (RT) is a critical part of breast-conservation therapy, shown to significantly improve relapse-free survival. The SweBCG91RT trial was a randomized phase III trial of adjuvant radiation which set this standard of care. Notably, very few patients in this trial received adjuvant systemic therapy (endocrine therapy or chemotherapy) and thus this dataset represents a unique opportunity to specifically identify CSTs associated with high risk of relapse after radiotherapy only. The majority of this trial was ER+, and so we focused on this subset of 590 patients, again noting that very few patients in this trial received adjuvant endocrine therapy. In order to identify patients who do poorly despite adjuvant RT, we focused on CSTs from SCAN-B where high expression (top quartile) was associated with worse recurrence rates, and also specifically in the RT-treated arm of SweBCG91RT (after multiple testing correction). Two CSTs were identified, *EPCAM* (Fig. 3A) and *CEACAM5* **(**Fig. 3B). Both were only associated with worse outcomes in the RT treated arm, not in the no RT arm, with the former having a statistically significant interaction with RT (*P* = 0.02), and the latter having a borderline significant interaction (*P* = 0.073). These results suggest that they may be associated specifically with worse radiation response, similar to what was seen for these targets in the SCAN-B cohort with post-endocrine therapy relapse. The interaction P-values remained similar after adjusting for tumor size, grade, and PAM50 (*P* = 0.022 and *P* = 0.071 respectively).

### CSTs expressed in HER2 + tumors at high risk of relapse after anti-HER2 therapies

**Fig. 4:**
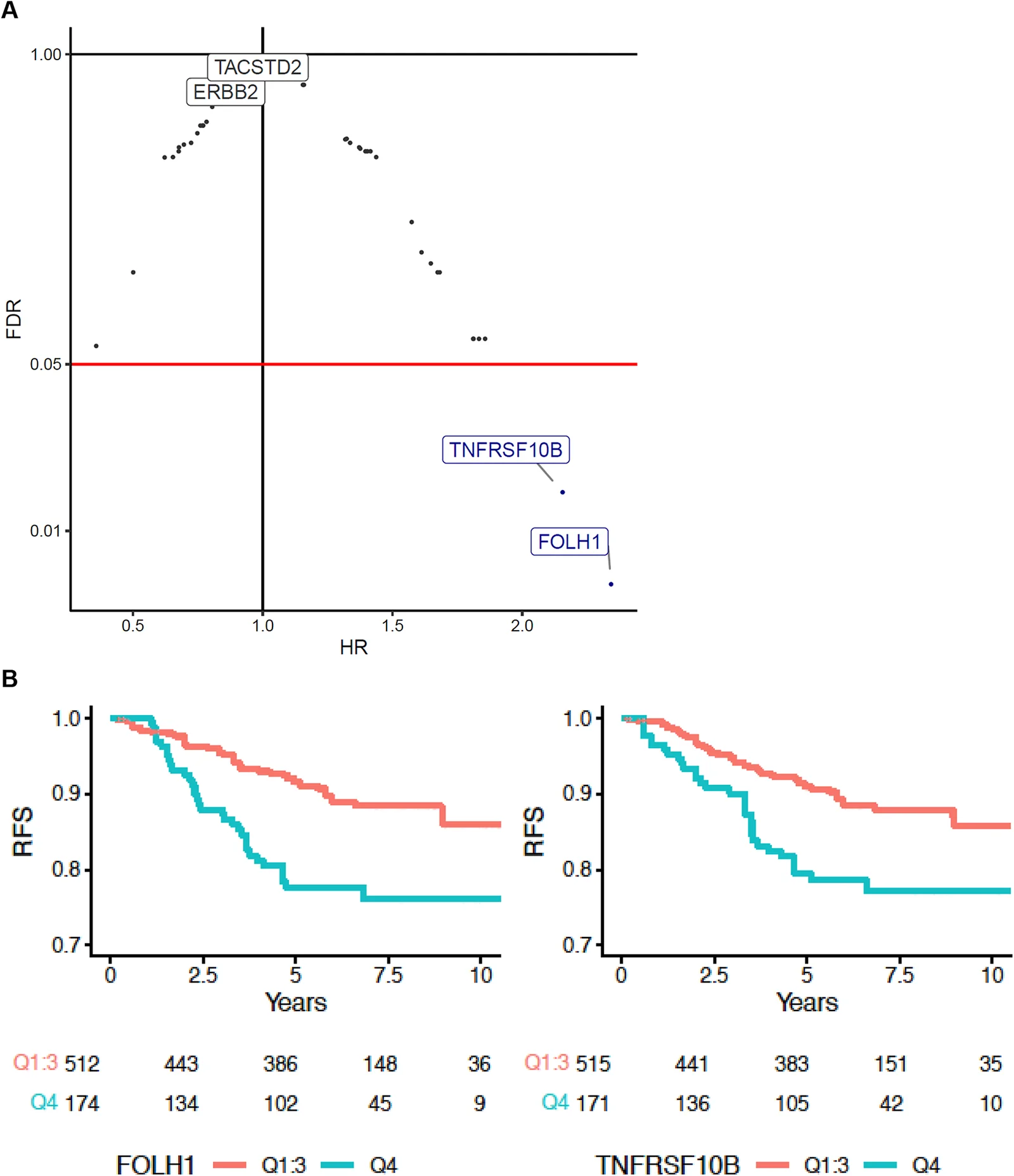
SCAN-B, HER2 + treated with anti-HER2 (*N* = 834). Volcano plot of the FDR vs. HR (**A**) and Kaplan-Meier curves (**B**) of top quartile (Q4) vs. bottom three quartiles (Q1:3) for each CST. FDR: False discovery rate, HR: hazard ratio, RFS: recurrence-free survival

The addition of anti-HER2 antibodies including trastuzumab and pertuzumab to chemotherapy has dramatically improved outcomes for patients with early-stage HER2-positive breast cancer, but metastatic relapses continue to drive morbidity and mortality associated with this breast cancer subtype. Patients with residual disease after neoadjuvant chemotherapy with anti-HER2 antibodies have relapse rates as high as 30–40% [47]. HER2-directed ADCs are known to reduce this risk, and in the randomized phase III KATHERINE trial, the HER2 ADC T-DM1 (trastuzumab emtansine) improved 7-year invasive disease-free survival from 67.1 to 80.8% in this group of patients [16], leading to FDA approval. More recently, a second generation HER2 ADC T-DXd (fam-trastuzumab deruxtecan) was reported to significantly improve invasive disease-free survival compared to T-DM1 in this patient population in the phase III DESTINY-Breast05 trial [15]. To identify novel targets and expand the spectrum of potential CST therapies for patients with HER2 + breast cancer at high risk of relapse after anti-HER2 therapies, we examined CST expression in 834 patients with HER2 + breast cancer who received anti-HER2 therapies in the SCAN-B cohort. As with ER+ breast cancer, neither *ERBB2* (HER2) nor *TACSTD2* (TROP2), the targets for existing FDA-approved ADCs in breast cancer, were associated with worse RFS (Fig. 4A). However, we did identify expression of two CSTs, *FOLH1* (PSMA) and *TNFRSF10B*, that were associated with markedly worse RFS in this cohort (Fig. 4B). These remained significantly associated after adjusting for clinicopathologic variables (age, nodal status, grade, pT-stage, PAM50; Table S4).

PSMA, the cell surface protein encoded by *FOLH1*, was first identified as a tumor CST in metastatic prostate cancer, where it is highly expressed, and serves as the target for the FDA-approved radioligand therapy 177Lu-PSMA-617 as well as the companion diagnostic radiotracer 68Ga-PSMA-11 [40]. However, PSMA protein is commonly detected in human HER2 + breast tumors and tumor microvasculature [3, 54]. A recent pilot study of 68Ga-PSMA-11 PET/CT in patients with metastatic breast cancer found that patients with HER2 + disease were more likely to have strong PSMA expression by this modality as well [31]. However, an association between high *FOLH1* expression and relapse after anti-HER2 therapy in HER2 + breast cancer has not previously been reported and suggests for the first time a potential role for repositioning of PSMA-targeted therapies for this patient population.

The *TNFRSF10B* gene encodes the protein TRAILR2, sometimes called DR5, which induces apoptosis and has been identified in human HER2 + breast tumors [13]. Several studies have sought to overcome trastuzumab resistance through TRAILR2/DR5 targeting in HER2 + breast cancer cells [19, 22, 53]. Interestingly anti-DR5 and anti-HER2 antibodies have been combined effectively in a HER2 + murine tumor model, leading to reduced drug resistance attributed to an immune response to the excessive cell death [47].

### CSTs associated with poor response to neoadjuvant chemotherapy in triple negative breast cancer

**Fig. 5:**
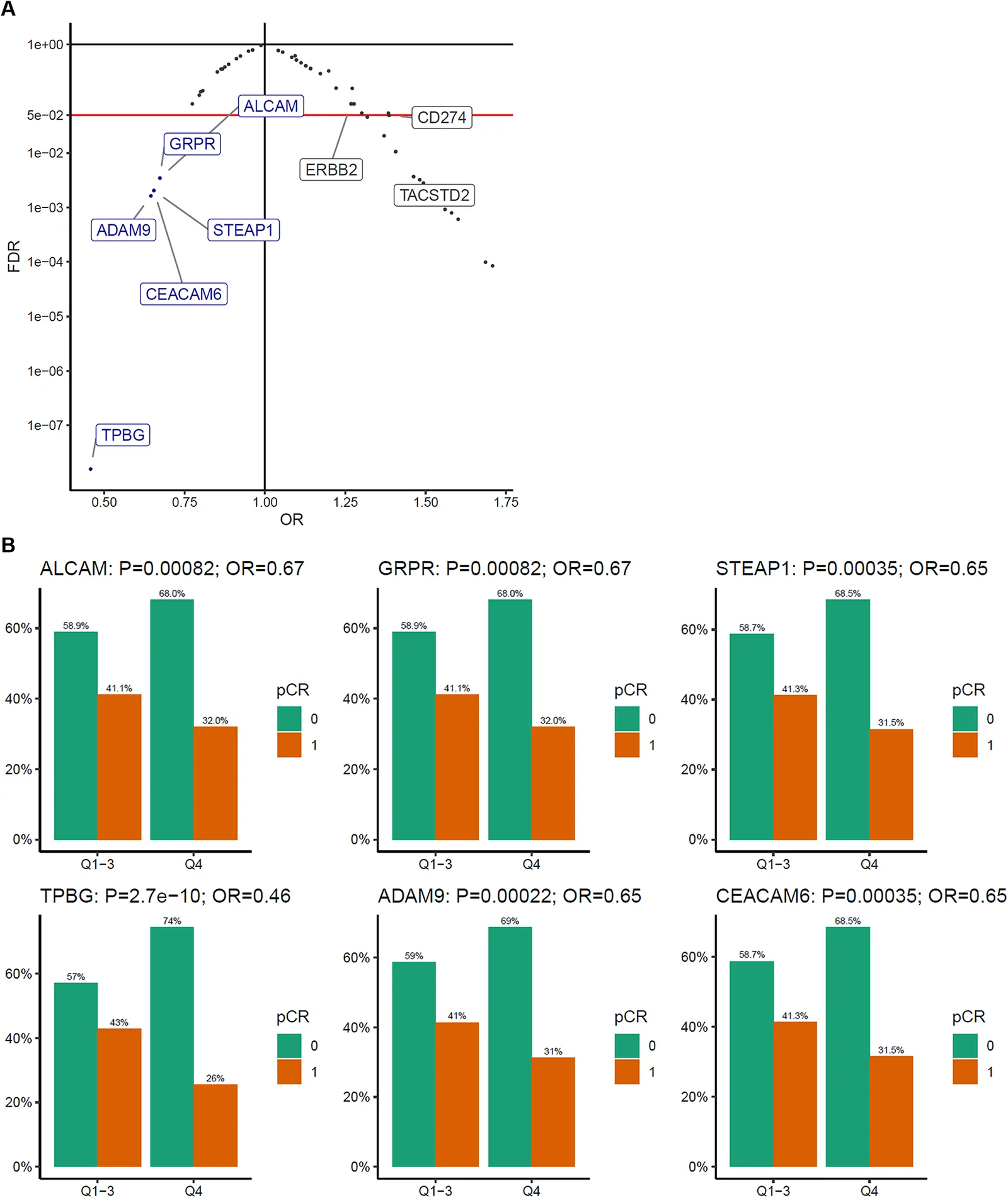
TNBC treated with neoadjuvant chemotherapy (*N* = 1686). Volcano plot of the FDR using Fisher’s exact test vs. OR (**A**) and barplots (**B**) of top quartile (Q4) vs. bottom three quartiles (Q1-3) for each CST. FDR: False discovery rate, OR: odds ratio, pCR: pathologic complete response (0 = no, 1 = yes)

As in early stage HER2 + breast cancer, residual disease after neoadjuvant chemotherapy or chemoimmunotherapy in triple negative breast cancer is associated with high risk of metastatic relapse and poor survival [10]. Median survival after a diagnosis of metastatic TNBC is less than 24 months [11], and there is an urgent need for therapies to reduce the risk of metastatic relapse in patients with residual disease. To evaluate for CSTs associated with residual disease in TNBC, we leveraged a dataset of pre-treatment gene expression data from 1686 patients who received neoadjuvant chemotherapy or chemoimmunotherapy for TNBC [5]. Consistent with previous reports [41], we found that *CD274* (PD-L1) expression was significantly associated with pathologic complete response (Fig. 5A). We also found that *ERBB2* (HER2) and *TACSTD2* (TROP2), the targets for existing FDA-approved ADCs in breast cancer that are currently being studied as adjuvant therapy for patients with TNBC residual disease [33, 50] were not associated with higher risk of residual disease, and in fact were significantly associated with pathologic complete response similar to *CD274* (Fig. 5A). In contrast, we identified 6 CSTs in pre-treatment samples that were significantly associated with subsequent residual disease after neoadjuvant therapy (Fig. 5A-B). Protein expression of 5/6 CSTs has been reported in at least a subset of human TNBC (*ALCAM* (CD166) [2, 8], *GRPR* [28], *STEAP1* [55], *ADAM9* [57], *CEACAM6* [51]) though prognostic associations for these CSTs within TNBC have not been previously defined. Thus, our identification of this group of CSTs enriched in tumors more likely to have residual disease after neoadjuvant therapy provides novel insights into the selection of drug repositioning candidates in this space where more effective therapies are sorely needed.

### Intratumoral CST expression heterogeneity across targets and breast cancer subtypes

**Fig. 6:**
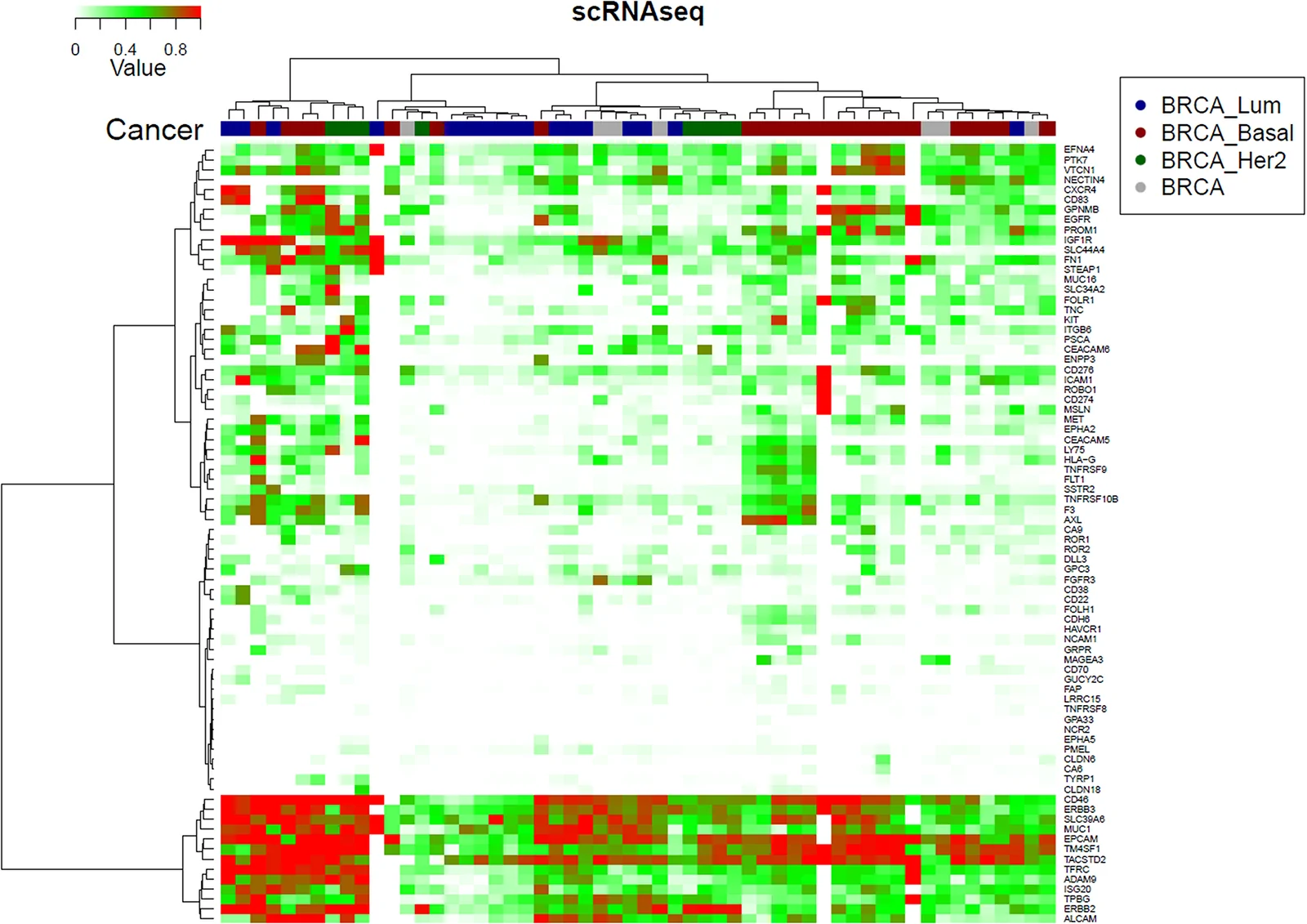
Heatmap of the proportion of the tumor cells in each scRNAseq breast cancer sample that expresses each CST. Rows are CSTs and columns are scRNAseq breast cancer samples. Red represents samples with a high proportion of tumor cells expressing each CST, green represents samples where a more mixed proportion of tumor cells express each CST, and white represents an absence of expression

The level and heterogeneity of a target’s expression within a tumor can impact the type of payload most likely to be effective. For example, a cytotoxic payload with hydrolyzable linker or a radiopharmaceutical lead to cell death in a region surrounding the target-expressing cell, while immunomodulatory payloads may require target engagement for each cell death event. We took advantage of scRNAseq data from 56 breast cancers to evaluate for intratumoral target heterogeneity of each of the 78 CSTs across breast cancer molecular subtypes (Fig. 6) and also in normal tissues [20, 43] (Figure S3). *EPCAM* and *TACSTD2* (TROP2), were the most uniformly expressed, but even so had a subset of samples with more heterogeneous expression. *ERBB2* (HER2), *ERBB3* (HER3), *ALCAM* and *ADAM9* were also consistently expressed in a subset of samples. In contrast, *CEACAM6 CEACAM5*,* NECTIN4*,* FN1*,* FOLH1*,* STEAP1 and TNFRSF10B* had fairly heterogeneous expression in the samples where they were detected, suggesting that these targets would be most appropriate for payloads with regional effects. None of these genes were strongly correlated with immune cell types (Figure S4).

## Discussion

ADCs have had early success in breast cancer, as both adjuvant therapy and in the metastatic setting, but FDA approved ADCs have focused on only two CSTs to date: *HER2* and *TROP2*, and there remains a need to identify novel CSTs in treatment-resistant breast cancer for therapeutic development, including potential repositioning of agents already under development for other tumor types. Most prior efforts have focused on identifying broadly expressed CSTs. In contrast, we sought to explore a complementary target-discovery strategy by identifying CSTs enriched in tumors with more aggressive clinical behavior and poorer outcomes. We reasoned that such targets may be particularly relevant for patient populations with substantial unmet clinical need. We leverage gene expression data from several large clinically annotated breast cancer cohorts and a randomized clinical trial to define for the first time the broader landscape of CST expression in treatment-resistant breast cancer across breast cancer subtypes and treatment types including both systemic therapies and radiation. Our clinically informed approach, identifying CSTs specifically associated with disease recurrence in early-stage breast cancer after standard therapies, gives us the unique opportunity to prioritize targets that are highly expressed in the clinical context where there is an unmet need for new treatment strategies.

We find that *HER2* and *TROP2* expression are not particularly associated with treatment resistant breast cancer, suggesting that agents targeting alternative CSTs may be needed to more effectively impact outcomes in this patient population. Indeed, we identify alternative CSTs associated with relapse after standard endocrine therapy in ER+ breast cancer, CSTs associated with relapse after radiation therapy in the absence of systemic therapy, and CSTs associated with poor outcomes in both HER2 + breast cancer and TNBC. Overlapping CST expression between some of these clinical settings, including association of *EPCAM*, *CEACAM5* with both endocrine and radiation resistant ER+ breast cancer, and *ADAM9*, *CEACAM6* with both endocrine resistant ER+ breast cancer and chemotherapy-resistant TNBC, suggests opportunities for aligned therapeutic development for treatment resistant disease, as well as the potential for conserved mechanisms that may drive association between CST expression and treatment resistance.

One limitation of our findings is the use of gene rather than protein expression to identify CSTs associated with treatment resistance. However, we have found that high gene expression is a good surrogate for protein expression across the CSTs in this study and others [43]. Additionally, most of the CSTs we identified as enriched in treatment resistant breast cancer have evidence of protein expression in published human breast cancer cohorts, and many have been confirmed to be associated with treatment resistance in other studies [1–4, 6–8, 12, 13, 19, 21–26, 28, 29, 31, 32, 34–38, 42, 47–49, 51, 53–57]. A second key limitation is that the degree of protein expression required for effective cell-surface targeted therapy is unknown and likely agent-dependent and the efficacy of CST therapies ultimately depend not only on target engagement but also linker-dependent payload delivery and tumor payload sensitivity.

Finally, as the landscape of available cell surface targeted therapies for breast cancer expands, biomarkers will be needed to select the appropriate therapy for each patient. Tissue availability and cost preclude high throughput screening of protein biomarkers via IHC, but as more commercial options emerge for tumor tissue RNA-seq in addition to DNA-seq for clinical use, RNA expression could potentially address this gap to identify candidate cell surface targets with high expression for subsequent confirmation by IHC [43].

## Supplementary information

Below is the link to the electronic supplementary material.

Supplementary Material 1

Supplementary Material 2

## Data Availability

All data have been previously published and were obtained via their respective publications described in the “Datasets” section in the methods.

## References

[1] Abd El-Maqsoud NM, Abd El-Rehim DM (2014) Clinicopathologic implications of EpCAM and Sox2 expression in breast cancer. Clin Breast Cancer 14:e1–9. 10.1016/j.clbc.2013.09.006 24201161

[2] Almasi S, Nagy A, Krenacs T, Lantos T, Zombori T, Cserni G (2023) The prognostic value of stem cell markers in triple-negative breast cancer. Pathol Oncol Res 29:1611365. 10.3389/pore.2023.1611365 PMC10766821 38188613

[3] Andryszak N, Kurzawa P, Krzyzaniak M, Nowicki M, Ruchala M, Izycki D, Czepczynski R (2024) Evaluation of Prostate-Specific Membrane Antigen (PSMA) immunohistochemical expression in early-stage breast cancer subtypes. Int J Mol Sci 25. 10.3390/ijms25126519 PMC11204143 38928224

[4] Arihiro K, Inai K, Kurihara K, Takeda S, Kaneko M (1993) Distribution of laminin, type IV collagen and fibronectin in the invasive component of breast carcinoma. Acta Pathol Jpn 43:758–764. 10.1111/j.1440-1827.1993.tb02563.x 8109253

[5] Bakhtiar H, Sharifi MN, Helzer KT, Shi Y, Bootsma ML, Shang TA, Chrostek MR, Berg TJ, Carson Callahan S, Carreno V, Blitzer GC, West MT, O’Regan RM, Wisinski KB, Sjostrom M, Zhao SG (2024) A phenocopy signature of TP53 loss predicts response to chemotherapy. NPJ Precis Oncol 8:220. 10.1038/s41698-024-00722-7 PMC11447220 39358429

[6] Balk-Moller E, Kim J, Hopkinson B, Timmermans-Wielenga V, Petersen OW, Villadsen R (2014) A marker of endocrine receptor-positive cells, CEACAM6, is shared by two major classes of breast cancer: luminal and HER2-enriched. Am J Pathol 184:1198–1208. 10.1016/j.ajpath.2013.12.013 24655379

[7] Bechmann MB, Brydholm AV, Codony VL, Kim J, Villadsen R (2020) Heterogeneity of CEACAM5 in breast cancer. Oncotarget 11:3886–3899. 10.18632/oncotarget.27778 PMC7597409 33196697

[8] Castaneda CA, Castillo M, Enciso JA, Enciso N, Bernabe LA, Sanchez J, Guerra H, Chavez C, Landa-Baella M, De-La-Cruz M, Villa-Robles M, Tello K, Gomez HL (2019) Role of undifferentiation markers and androgen receptor expression in triple-negative breast cancer. Breast J 25:1316–1319. 10.1111/tbj.13464 31332870

[9] Colleoni M, Sun Z, Price KN, Karlsson P, Forbes JF, Thurlimann B, Gianni L, Castiglione M, Gelber RD, Coates AS, Goldhirsch A (2016) Annual Hazard Rates of Recurrence for Breast Cancer During 24 Years of Follow-Up: Results From the International Breast Cancer Study Group Trials I to V. J Clin Oncol 34:927–935. 10.1200/JCO.2015.62.3504 PMC4933127 26786933

[10] Cortazar P, Zhang L, Untch M, Mehta K, Costantino JP, Wolmark N, Bonnefoi H, Cameron D, Gianni L, Valagussa P, Swain SM, Prowell T, Loibl S, Wickerham DL, Bogaerts J, Baselga J, Perou C, Blumenthal G, Blohmer J, Mamounas EP, Bergh J, Semiglazov V, Justice R, Eidtmann H, Paik S, Piccart M, Sridhara R, Fasching PA, Slaets L, Tang S, Gerber B, Geyer CE Jr., Pazdur R, Ditsch N, Rastogi P, Eiermann W, von Minckwitz G (2014) Pathological complete response and long-term clinical benefit in breast cancer: the CTNeoBC pooled analysis. Lancet 384:164–172. 10.1016/S0140-6736(13)62422-8 24529560

[11] Cortes J, Rugo HS, Cescon DW, Im SA, Yusof MM, Gallardo C, Lipatov O, Barrios CH, Perez-Garcia J, Iwata H, Masuda N, Torregroza Otero M, Gokmen E, Loi S, Guo Z, Zhou X, Karantza V, Pan W, Schmid P, Investigators K- (2022) Pembrolizumab plus Chemotherapy in Advanced Triple-Negative Breast Cancer. N Engl J Med 387:217–226. 10.1056/NEJMoa2202809

[12] Fang D, Lin J, Wang J, Nong Q, Tao S, Lu B, Yu Y, Peng H, Tian Y, Su Q, Ma Y, Huang Y (2025) CEACAM6 as a machine learning derived immune biomarker for predicting neoadjuvant chemotherapy response in HR+/HER2- breast cancer. Front Immunol 16:1662004. 10.3389/fimmu.2025.1662004 PMC12420257 40936892

[13] Ganten TM, Sykora J, Koschny R, Batke E, Aulmann S, Mansmann U, Stremmel W, Sinn HP, Walczak H (2009) Prognostic significance of tumour necrosis factor-related apoptosis-inducing ligand (TRAIL) receptor expression in patients with breast cancer. J Mol Med (Berl) 87:995–1007. 10.1007/s00109-009-0510-z 19680616

[14] Gavish A, Tyler M, Greenwald AC, Hoefflin R, Simkin D, Tschernichovsky R, Galili Darnell N, Somech E, Barbolin C, Antman T, Kovarsky D, Barrett T, Gonzalez Castro LN, Halder D, Chanoch-Myers R, Laffy J, Mints M, Wider A, Tal R, Spitzer A, Hara T, Raitses-Gurevich M, Stossel C, Golan T, Tirosh A, Suva ML, Puram SV, Tirosh I (2023) Hallmarks of transcriptional intratumour heterogeneity across a thousand tumours. Nature 618:598–606. 10.1038/s41586-023-06130-4 37258682

[15] Geyer CE, Park YH, Shao ZM, Huang CS, Barrios CHE, Abraham J, Prat A, Niikura N,Untch M, Im SA, Li W, Li H, Wang Y, Yao H, Kim SB, Mathias E, Sato Y, Lu W, Abdel-Monem H, Loibl S (2025) LBA1 Trastuzumab deruxtecan (T-DXd) vs trastuzumab emtansine (T-DM1)in patients (pts) with high-risk human epidermal growth factor receptor 2–positive(HER2+) primary breast cancer (BC) with residual invasive disease after neoadjuvant therapy (tx): Interim analysis of DESTINY-Breast05. Ann Oncol 36:S1671–S1672. 10.1016/j.annonc.2025.09.021

[16] Geyer CE Jr., Untch M, Huang CS, Mano MS, Mamounas EP, Wolmark N, Rastogi P, Schneeweiss A, Redondo A, Fischer HH, D’Hondt V, Conlin AK, Guarneri V, Wapnir IL, Jackisch C, Arce-Salinas C, Fasching PA, DiGiovanna MP, Crown JP, Wuelfing P, Shao Z, Rota Caremoli E, Bonnefoi HR, Hennessy BT, Stamatovic L, Castro-Salguero H, Brufsky AM, Knott A, Siddiqui A, Lambertini C, Boulet T, Nyawira B, Restuccia E, Loibl S, Group KS (2025) Survival with Trastuzumab Emtansine in Residual HER2-Positive Breast Cancer. N Engl J Med 392:249–257. 10.1056/NEJMoa2406070 39813643

[17] Giaquinto AN, Sung H, Newman LA, Freedman RA, Smith RA, Star J, Jemal A, Siegel RL (2024) Breast cancer statistics 2024. CA Cancer J Clin 74:477–495. 10.3322/caac.21863 39352042

[18] Goncalves E, Poulos RC, Cai Z, Barthorpe S, Manda SS, Lucas N, Beck A, Bucio-Noble D, Dausmann M, Hall C, Hecker M, Koh J, Lightfoot H, Mahboob S, Mali I, Morris J, Richardson L, Seneviratne AJ, Shepherd R, Sykes E, Thomas F, Valentini S, Williams SG, Wu Y, Xavier D, MacKenzie KL, Hains PG, Tully B, Robinson PJ, Zhong Q, Garnett MJ, Reddel RR (2022) Pan-cancer proteomic map of 949 human cell lines. Cancer Cell 40:835–849e838. 10.1016/j.ccell.2022.06.010 PMC9387775 35839778

[19] Greer YE, Gilbert SF, Gril B, Narwal R, Peacock Brooks DL, Tice DA, Steeg PS, Lipkowitz S (2019) MEDI3039, a novel highly potent tumor necrosis factor (TNF)-related apoptosis-inducing ligand (TRAIL) receptor 2 agonist, causes regression of orthotopic tumors and inhibits outgrowth of metastatic triple-negative breast cancer. Breast Cancer Res 21:27. 10.1186/s13058-019-1116-1 PMC6380056 30777098

[20] Han X, Zhou Z, Fei L, Sun H, Wang R, Chen Y, Chen H, Wang J, Tang H, Ge W, Zhou Y, Ye F, Jiang M, Wu J, Xiao Y, Jia X, Zhang T, Ma X, Zhang Q, Bai X, Lai S, Yu C, Zhu L, Lin R, Gao Y, Wang M, Wu Y, Zhang J, Zhan R, Zhu S, Hu H, Wang C, Chen M, Huang H, Liang T, Chen J, Wang W, Zhang D, Guo G (2020) Construction of a human cell landscape at single-cell level. Nature 581:303–309. 10.1038/s41586-020-2157-4 32214235

[21] Lattanzio R, Ghasemi R, Brancati F, Sorda RL, Tinari N, Perracchio L, Iacobelli S, Mottolese M, Natali PG, Piantelli M (2014) Membranous Nectin-4 expression is a risk factor for distant relapse of T1-T2, N0 luminal-A early breast cancer. Oncogenesis 3:e118. 10.1038/oncsis.2014.32 PMC4183981 25178039

[22] Law ME, Yaaghubi E, Ghilardi AF, Davis BJ, Ferreira RB, Koh J, Chen S, DePeter SF, Schilson CM, Chiang CW, Heldermon CD, Norgaard P, Castellano RK, Law BK (2022) Inhibitors of ERp44, PDIA1, and AGR2 induce disulfide-mediated oligomerization of Death Receptors 4 and 5 and cancer cell death. Cancer Lett 534:215604. 10.1016/j.canlet.2022.215604 35247515

[23] Lebok P, Huber J, Burandt EC, Lebeau A, Marx AH, Terracciano L, Heilenkotter U, Janicke F, Muller V, Paluchowski P, Geist S, Wilke C, Simon R, Sauter G, Quaas A (2016) Loss of membranous VEGFR1 expression is associated with an adverse phenotype and shortened survival in breast cancer. Mol Med Rep 14:1443–1450. 10.3892/mmr.2016.5430 PMC4940099 27357606

[24] Lee DW, Ryu HS, Jin MS, Lee KH, Suh KJ, Youk J, Kim JY, Min A, Lee HB, Moon HG, Kim TY, Han SW, Oh DY, Han W, Park IA, Noh DY, Im SA (2019) Immune recurrence score using 7 immunoregulatory protein expressions can predict recurrence in stage I-III breast cancer patients. Br J Cancer 121:230–236. 10.1038/s41416-019-0511-9 PMC6738097 31292534

[25] Lewis-Wambi JS, Cunliffe HE, Kim HR, Willis AL, Jordan VC (2008) Overexpression of CEACAM6 promotes migration and invasion of oestrogen-deprived breast cancer cells. Eur J Cancer 44:1770–1779. 10.1016/j.ejca.2008.05.016 PMC2778047 18614350

[26] Maraqa L, Cummings M, Peter MB, Shaaban AM, Horgan K, Hanby AM, Speirs V (2008) Carcinoembryonic antigen cell adhesion molecule 6 predicts breast cancer recurrence following adjuvant tamoxifen. Clin Cancer Res 14:405–411. 10.1158/1078-0432.CCR-07-1363 18223215

[27] Marczyk VR, Rosa DD, Maia AL, Goemann IM (2022) Overall Survival for HER2-Positive Breast Cancer Patients in the HER2-Targeted Era: Evidence From a Population-Based Study. Clin Breast Cancer 22:418–423. 10.1016/j.clbc.2022.03.004 35430163

[28] Morgat C, MacGrogan G, Brouste V, Velasco V, Sevenet N, Bonnefoi H, Fernandez P, Debled M, Hindie E (2017) Expression of Gastrin-Releasing Peptide Receptor in Breast Cancer and Its Association with Pathologic, Biologic, and Clinical Parameters: A Study of 1,432 Primary Tumors. J Nucl Med 58:1401–1407. 10.2967/jnumed.116.188011 28280221

[29] Nafissi N, Azad Armaki S, Babaee E, Babaheidarian P, Safari E, Sayad S, Saghafinia S, Safaee M (2024) Association between EPCAM upregulation and clinicopathological parameters and outcomes of breast cancer. Int J Clin Exp Pathol 17:421–428. 10.62347/EGXS1506 PMC11626292 39660329

[30] Pan H, Gray R, Braybrooke J, Davies C, Taylor C, McGale P, Peto R, Pritchard KI, Bergh J, Dowsett M, Hayes DF, Ebctcg (2017) 20-Year Risks of Breast-Cancer Recurrence after Stopping Endocrine Therapy at 5 Years. N Engl J Med 377:1836–1846. 10.1056/NEJMoa1701830 PMC5734609 29117498

[31] Parghane RV, Suralkar T, Nikam D, Basu S (2024) Evaluation of prostate-specific membrane antigen expression in locally advanced or metastatic breast carcinoma with 68 Ga-PSMA-11 positron-emission tomography/computed tomography imaging for potential theranostics. Nucl Med Commun 45:329–337. 10.1097/MNM.0000000000001820 38312049

[32] Penrod NM, Greene CS, Moore JH (2014) Predicting targeted drug combinations based on Pareto optimal patterns of coexpression network connectivity. Genome Med 6:33. 10.1186/gm550 PMC4062052 24944582

[33] Pfister K, Friedl TWP, Hartkopf A, Mergel F, Huesmann S, Mehmeti F, Schaffler H, Fink A, Braun T, Heublein S, Wiesmuller L, Pantel K, Rack B, Fasching PA, Janni W (2025) Study Protocol of SURVIVE HERoes (NCT06643585): Trastuzumab Deruxtecan for molecular relapse in HER2+/ Low early breast cancer with ctDNA positivity after primary therapy. PLoS ONE 20:e0322156. 10.1371/journal.pone.0322156 PMC12629461 41259337

[34] Pontiggia O, Sampayo R, Raffo D, Motter A, Xu R, Bissell MJ, Joffe EB, Simian M (2012) The tumor microenvironment modulates tamoxifen resistance in breast cancer: a role for soluble stromal factors and fibronectin through beta1 integrin. Breast Cancer Res Treat 133:459–471. 10.1007/s10549-011-1766-x PMC3719875 21935603

[35] Rajc J, Gugic D, Frohlich I, Marjanovic K, Dumencic B (2017) Prognostic role of Nectin-4 expression in luminal B (HER2 negative) breast cancer. Pathol Res Pract 213:1102–1108. 10.1016/j.prp.2017.07.019 28778498

[36] Ring BZ, Seitz RS, Beck R, Shasteen WJ, Tarr SM, Cheang MC, Yoder BJ, Budd GT, Nielsen TO, Hicks DG, Estopinal NC, Ross DT (2006) Novel prognostic immunohistochemical biomarker panel for estrogen receptor-positive breast cancer. J Clin Oncol 24:3039–3047. 10.1200/JCO.2006.05.6564 16809728

[37] Roll JD, Rivenbark AG, Sandhu R, Parker JS, Jones WD, Carey LA, Livasy CA, Coleman WB (2013) Dysregulation of the epigenome in triple-negative breast cancers: basal-like and claudin-low breast cancers express aberrant DNA hypermethylation. Exp Mol Pathol 95:276–287. 10.1016/j.yexmp.2013.09.001 24045095

[38] Sampayo RG, Toscani AM, Rubashkin MG, Thi K, Masullo LA, Violi IL, Lakins JN, Caceres A, Hines WC, Coluccio Leskow F, Stefani FD, Chialvo DR, Bissell MJ, Weaver VM, Simian M (2018) Fibronectin rescues estrogen receptor alpha from lysosomal degradation in breast cancer cells. J Cell Biol 217:2777–2798. 10.1083/jcb.201703037 PMC6080927 29980625

[39] Saphner T, Tormey DC, Gray R (1996) Annual hazard rates of recurrence for breast cancer after primary therapy. J Clin Oncol 14:2738–2746. 10.1200/JCO.1996.14.10.2738 8874335

[40] Sartor O, de Bono J, Chi KN, Fizazi K, Herrmann K, Rahbar K, Tagawa ST, Nordquist LT, Vaishampayan N, El-Haddad G, Park CH, Beer TM, Armour A, Perez-Contreras WJ, DeSilvio M, Kpamegan E, Gericke G, Messmann RA, Morris MJ, Krause BJ, Investigators V (2021) Lutetium-177-PSMA-617 for Metastatic Castration-Resistant Prostate Cancer. N Engl J Med 385:1091–1103. 10.1056/NEJMoa2107322 PMC8446332 34161051

[41] Schmid P, Cortes J, Pusztai L, McArthur H, Kummel S, Bergh J, Denkert C, Park YH, Hui R, Harbeck N, Takahashi M, Foukakis T, Fasching PA, Cardoso F, Untch M, Jia L, Karantza V, Zhao J, Aktan G, Dent R, O’Shaughnessy J, Investigators K- (2020) Pembrolizumab for Early Triple-Negative Breast Cancer. N Engl J Med 382:810–821. 10.1056/NEJMoa1910549

[42] Schmidt M, Hasenclever D, Schaeffer M, Boehm D, Cotarelo C, Steiner E, Lebrecht A, Siggelkow W, Weikel W, Schiffer-Petry I, Gebhard S, Pilch H, Gehrmann M, Lehr HA, Koelbl H, Hengstler JG, Schuler M (2008) Prognostic effect of epithelial cell adhesion molecule overexpression in untreated node-negative breast cancer. Clin Cancer Res 14:5849–5855. 10.1158/1078-0432.CCR-08-0669 18794096

[43] Sharifi MN, Shi Y, Chrostek MR, Callahan SC, Shang T, Berg TJ, Helzer KT, Bootsma ML, Sjostrom M, Josefsson A, Feng FY, Huffman LB, Schulte C, Blitzer GC, Sodji QH, Morris ZS, Ma VT, Meimetis L, Kosoff D, Taylor AK, LeBeau AM, Lang JM, Zhao SG (2024) Clinical cell-surface targets in metastatic and primary solid cancers. JCI Insight 9. 10.1172/jci.insight.183674 PMC11457844 39315546

[44] Sjostrom M, Chang SL, Fishbane N, Davicioni E, Zhao SG, Hartman L, Holmberg E, Feng FY, Speers CW, Pierce LJ, Malmstrom P, Ferno M, Karlsson P (2019) Clinicogenomic Radiotherapy Classifier Predicting the Need for Intensified Locoregional Treatment After Breast-Conserving Surgery for Early-Stage Breast Cancer. J Clin Oncol 37:3340–3349. 10.1200/JCO.19.00761 PMC6901281 31618132

[45] Sjostrom M, Fyles A, Liu FF, McCready D, Shi W, Rey-McIntyre K, Chang SL, Feng FY, Speers CW, Pierce LJ, Holmberg E, Ferno M, Malmstrom P, Karlsson P (2023) Development and Validation of a Genomic Profile for the Omission of Local Adjuvant Radiation in Breast Cancer. J Clin Oncol 41:1533–1540. 10.1200/JCO.22.00655 PMC10022846 36599119

[46] Staaf J, Hakkinen J, Hegardt C, Saal LH, Kimbung S, Hedenfalk I, Lien T, Sorlie T, Naume B, Russnes H, Marcone R, Ayyanan A, Brisken C, Malterling RR, Asking B, Olofsson H, Lindman H, Bendahl PO, Ehinger A, Larsson C, Loman N, Ryden L, Malmberg M, Borg A, Vallon-Christersson J (2022) RNA sequencing-based single sample predictors of molecular subtype and risk of recurrence for clinical assessment of early-stage breast cancer. NPJ Breast Cancer 8:94. 10.1038/s41523-022-00465-3 PMC9381586 35974007

[47] Stagg J, Sharkey J, Pommey S, Young R, Takeda K, Yagita H, Johnstone RW, Smyth MJ (2008) Antibodies targeted to TRAIL receptor-2 and ErbB-2 synergize in vivo and induce an antitumor immune response. Proc Natl Acad Sci U S A 105:16254–16259. 10.1073/pnas.0806849105 PMC2570981 18838682

[48] Suman S, Basak T, Gupta P, Mishra S, Kumar V, Sengupta S, Shukla Y (2016) Quantitative proteomics revealed novel proteins associated with molecular subtypes of breast cancer. J Proteom 148:183–193. 10.1016/j.jprot.2016.07.033 27498393

[49] Tan Q, Xu L, Zhang J, Ning L, Jiang Y, He T, Luo J, Chen J, Lv Q, Yang X, Xie H (2023) Breast cancer cells interact with tumor-derived extracellular matrix in a molecular subtype-specific manner. Biomater Adv 146:213301. 10.1016/j.bioadv.2023.213301 36709629

[50] Tolaney SM, DeMichele A, Takano T, Rugo HS, Perou C, Lynce F, Parsons HA, Santa-Maria CA, Rocque GB, Yao W, Sun SW, Mocci S, Partridge AH, Carey LA (2024) OptimICE-RD: sacituzumab govitecan + pembrolizumab vs pembrolizumab (+/- capecitabine) for residual triple-negative breast cancer. Future Oncol 20:2343–2355. 10.1080/14796694.2024.2357534 PMC11520537 38922307

[51] Tsang JY, Kwok YK, Chan KW, Ni YB, Chow WN, Lau KF, Shao MM, Chan SK, Tan PH, Tse GM (2013) Expression and clinical significance of carcinoembryonic antigen-related cell adhesion molecule 6 in breast cancers. Breast Cancer Res Treat 142:311–322. 10.1007/s10549-013-2756-y 24186057

[52] Vici P, Pizzuti L, Natoli C, Moscetti L, Mentuccia L, Vaccaro A, Sergi D, Di Lauro L, Trenta P, Seminara P, Santini D, Iezzi L, Tinari N, Bertolini I, Sini V, Mottolese M, Giannarelli D, Giotta F, Maugeri-Sacca M, Barba M, Marchetti P, Michelotti A, Sperduti I, Gamucci T (2014) Outcomes of HER2-positive early breast cancer patients in the pre-trastuzumab and trastuzumab eras: a real-world multicenter observational analysis. The RETROHER study. Breast Cancer Res Treat 147:599–607. 10.1007/s10549-014-3133-1 PMC4174332 25234842

[53] Wang M, Law ME, Davis BJ, Yaaghubi E, Ghilardi AF, Ferreira RB, Chiang CW, Guryanova OA, Kopinke D, Heldermon CD, Castellano RK, Law BK (2019) Disulfide bond-disrupting agents activate the tumor necrosis family-related apoptosis-inducing ligand/death receptor 5 pathway. Cell Death Discov 5:153. 10.1038/s41420-019-0228-9 PMC6904486 31839995

[54] Wernicke AG, Varma S, Greenwood EA, Christos PJ, Chao KS, Liu H, Bander NH, Shin SJ (2014) Prostate-specific membrane antigen expression in tumor-associated vasculature of breast cancers. APMIS 122:482–489. 10.1111/apm.12195 24304465

[55] Xie J, Yang Y, Sun J, Jiao Z, Zhang H, Chen J (2019) STEAP1 Inhibits Breast Cancer Metastasis and Is Associated With Epithelial-Mesenchymal Transition Procession. Clin Breast Cancer 19:e195–e207. 10.1016/j.clbc.2018.08.010 30253922

[56] Yuan J, Liu M, Yang L, Tu G, Zhu Q, Chen M, Cheng H, Luo H, Fu W, Li Z, Yang G (2015) Acquisition of epithelial-mesenchymal transition phenotype in the tamoxifen-resistant breast cancer cell: a new role for G protein-coupled estrogen receptor in mediating tamoxifen resistance through cancer-associated fibroblast-derived fibronectin and beta1-integrin signaling pathway in tumor cells. Breast Cancer Res 17:69. 10.1186/s13058-015-0579-y PMC4453053 25990368

[57] Zhou R, Cho WCS, Ma V, Cheuk W, So YK, Wong SCC, Zhang M, Li C, Sun Y, Zhang H, Chan LWC, Tian M (2020) ADAM9 Mediates Triple-Negative Breast Cancer Progression via AKT/NF-kappaB Pathway. Front Med (Lausanne) 7:214. 10.3389/fmed.2020.00214 PMC7317048 32637415

