# Supplementary material for: The landscape of cell surface target expression in treatment resistant early-stage breast cancer
Source: Breast Cancer Res Treat. 2026 Nov 9;220(1):34. doi: 10.1007/s10549-026-08120-2 (PMC13748169; doi:10.1007/s10549-026-08120-2)
Supplement: Supplementary file 1 [file 10549_2026_8120_MOESM1_ESM.pdf]

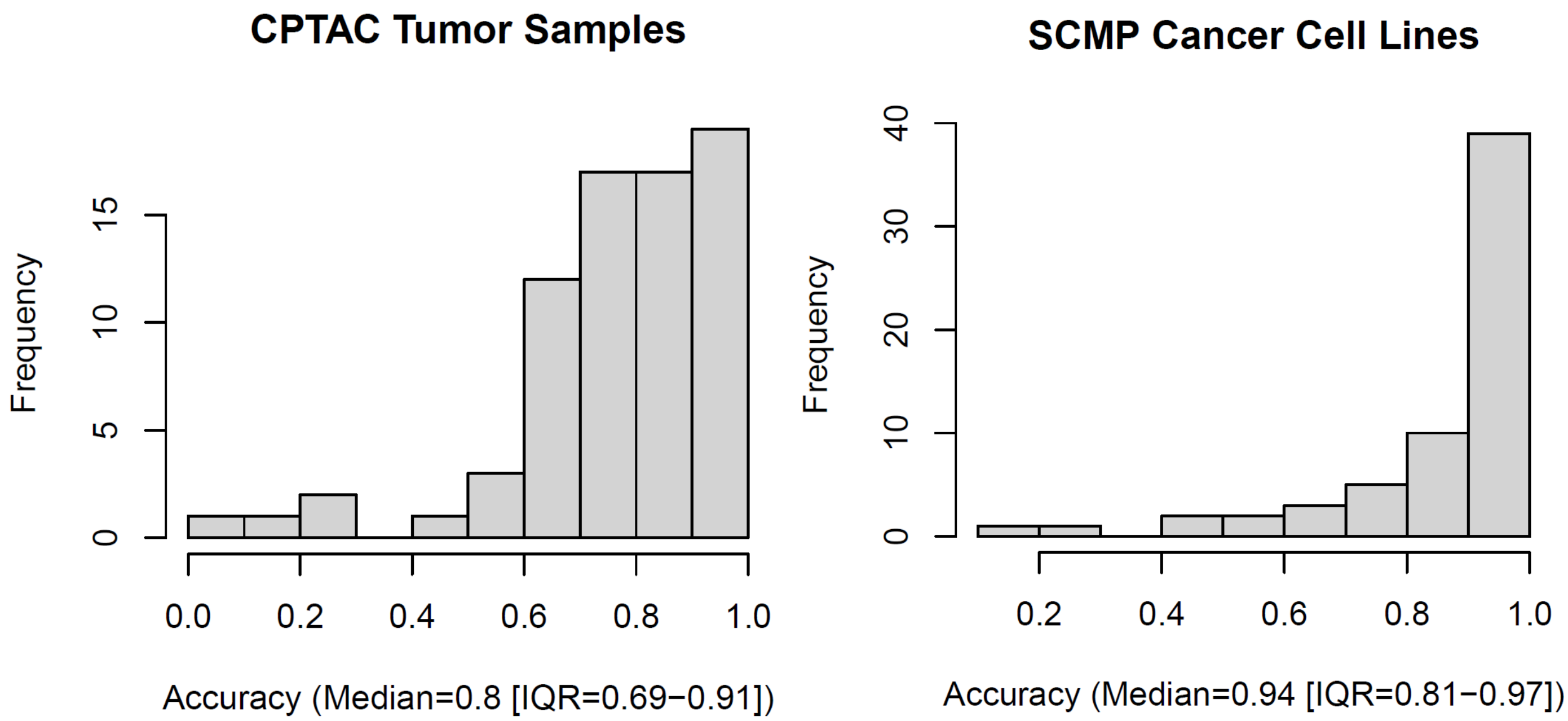

Figure S1: RNA vs. protein expression in two datasets with matched RNA-seq and mass spectrometry: cancer tissue samples from the Clinical Proteomic Tumor Analysis Consortium (CPTAC), cancer cell lines from the Sanger Cell Model Passports (SCMP). We defined high expression of protein or RNA as being higher than the 75th percentile of all genes in each sample. Histograms showing the distribution of accuracies of high RNA predicting high protein expression across cell surface targets comparing protein and RNA are shown.

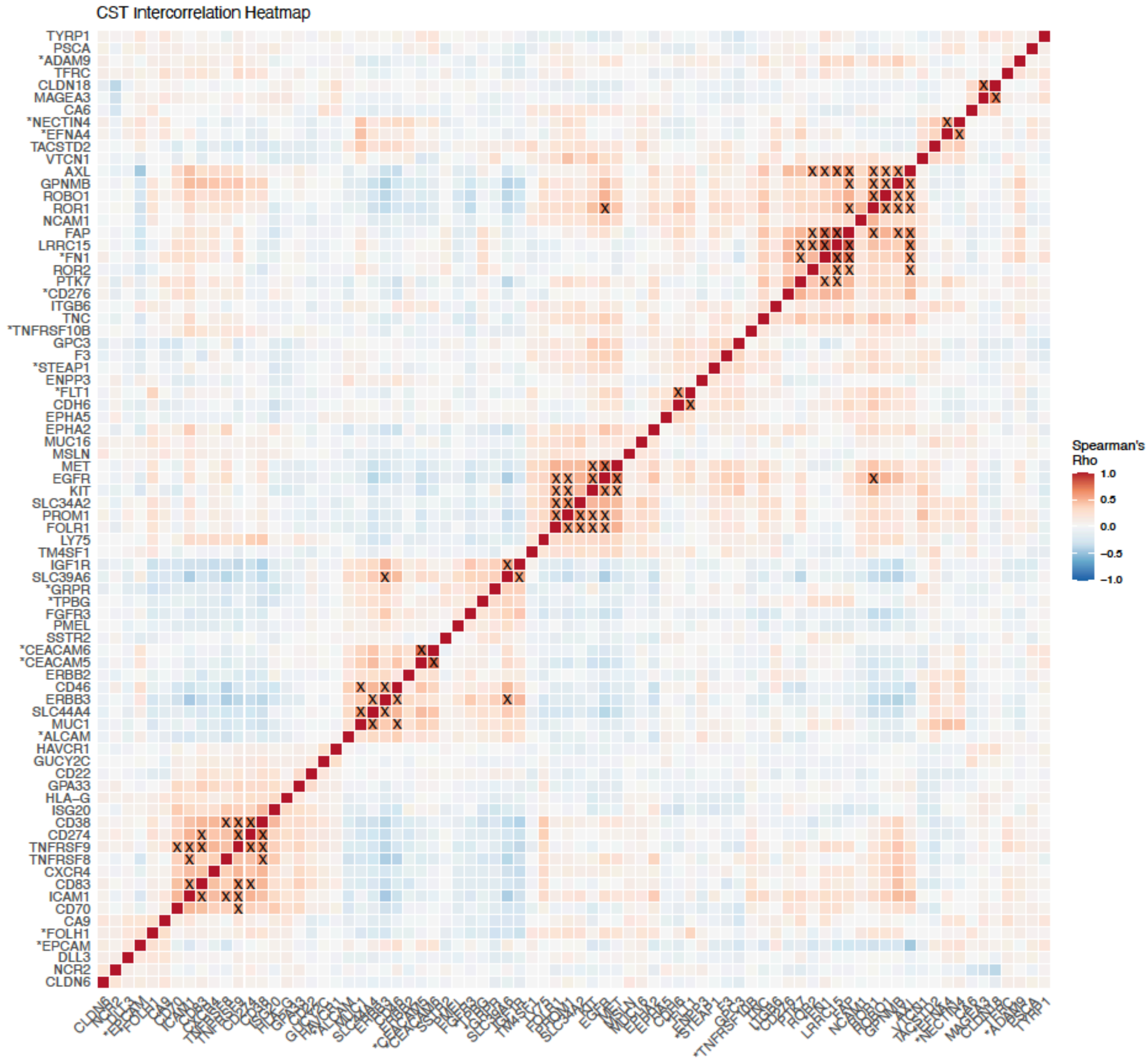

Figure S2: Heatmap of Spearman's correlation in SCAN-B of all CSTs. *CSTs highlighted in the manuscript. X indicates a Spearman's correlation >0.5.

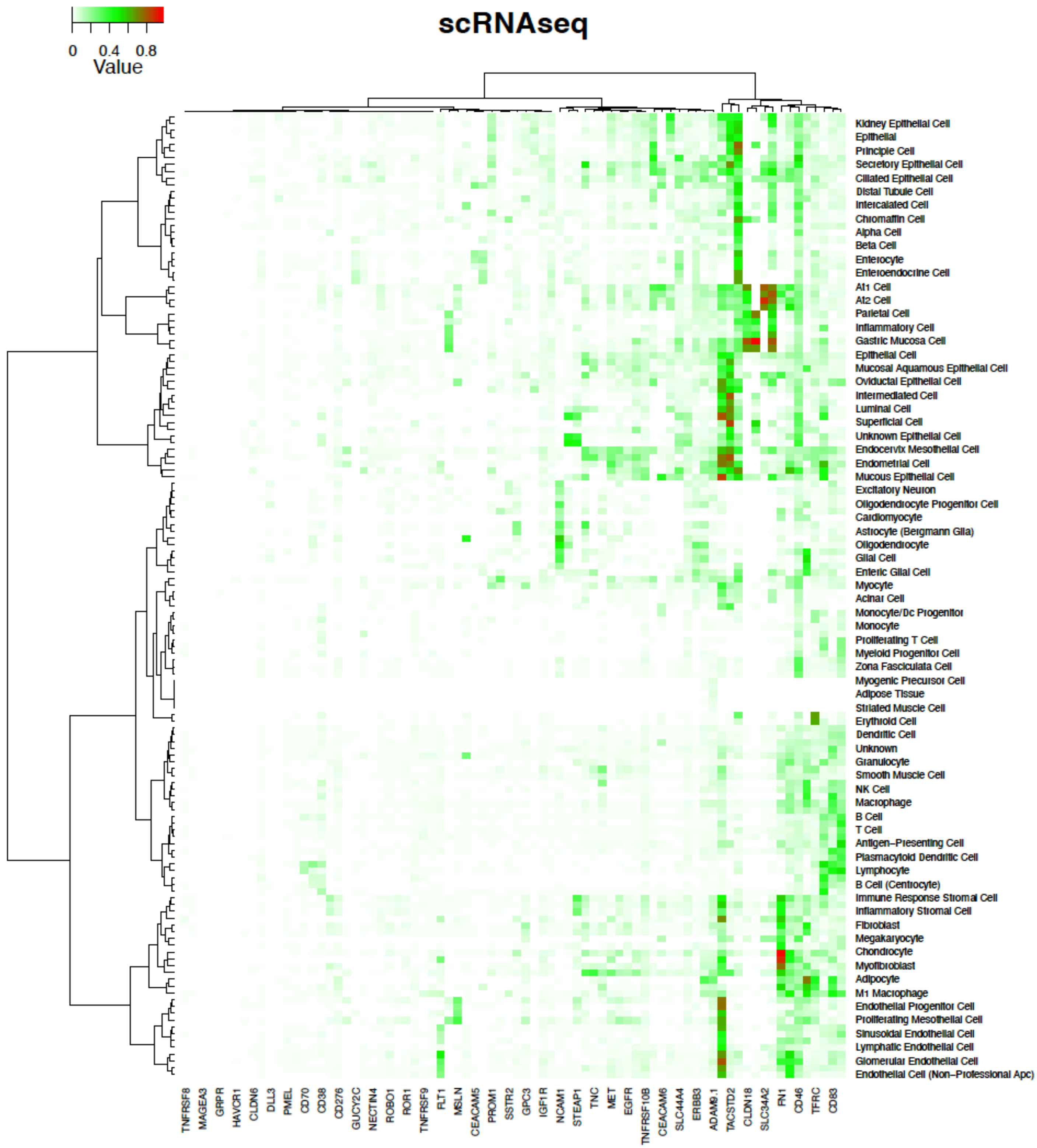

Figure S3: Heatmap of the proportion of the cells in each scRNAseq non-cancer cell type that expresses each CST. Rows are CSTs and columns are scRNAseq non-cancer cells. Red represents samples with a high proportion of cells expressing each CST, green represents samples where a more mixed proportion of cells express each CST, and white represents an absence of expression.

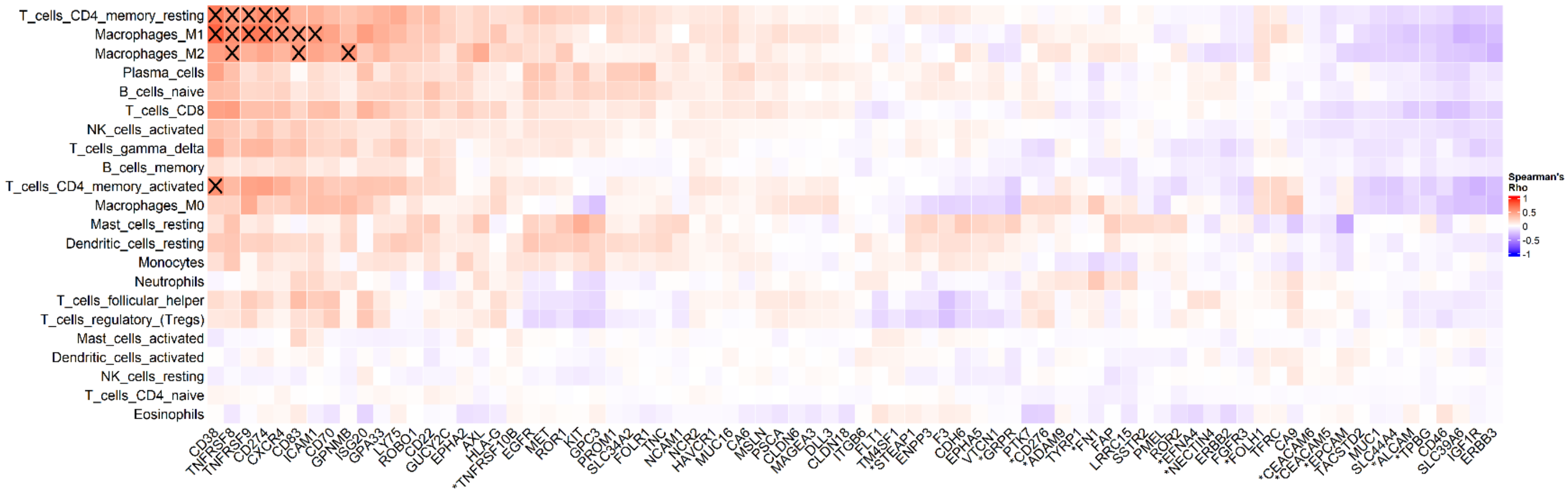

Figure S4: Heatmap of Spearman's correlation in SCAN-B of all CSTs with deconvolved immune subsets using the CIBERSORT algorithm with the validated LM22 signature matrix. *CSTs highlighted in the manuscript. X indicates a Spearman's correlation >0.5.
